# *Chlamydia trachomatis* Infection of Endocervical Epithelial Cells Enhances Early HIV Transmission Events

**DOI:** 10.1371/journal.pone.0146663

**Published:** 2016-01-05

**Authors:** Lyndsey R. Buckner, Angela M. Amedee, Hannah L. Albritton, Pamela A. Kozlowski, Nedra Lacour, Chris L. McGowin, Danny J. Schust, Alison J. Quayle

**Affiliations:** 1 Department of Microbiology, Immunology and Parasitology, Louisiana State University Health Sciences Center, New Orleans, Louisiana 70112, United States of America; 2 Department of Medicine, Section of Infectious Diseases, Louisiana State University Health Sciences Center, New Orleans, Louisiana, 70112, United States of America; 3 Department of Obstetrics, Gynecology and Women’s Health, University of Missouri, Columbia, MO 65201, United States of America; University of California, San Francisco, University of California, Berkeley, and the Children's Hospital Oakland Research Institute, UNITED STATES

## Abstract

*Chlamydia trachomatis* causes a predominantly asymptomatic, but generally inflammatory, genital infection that is associated with an increased risk for HIV acquisition. Endocervical epithelial cells provide the major niche for this obligate intracellular bacterium in women, and the endocervix is also a tissue in which HIV transmission can occur. The mechanism by which CT infection enhances HIV susceptibility at this site, however, is not well understood. Utilizing the A2EN immortalized endocervical epithelial cell line grown on cell culture inserts, we evaluated the direct role that CT-infected epithelial cells play in facilitating HIV transmission events. We determined that CT infection significantly enhanced the apical-to-basolateral migration of cell-associated, but not cell-free, HIV_BaL_, a CCR5-tropic strain of virus, across the endocervical epithelial barrier. We also established that basolateral supernatants from CT-infected A2EN cells significantly enhanced HIV replication in peripheral mononuclear cells and a CCR5+ T cell line. These results suggest that CT infection of endocervical epithelial cells could facilitate both HIV crossing the mucosal barrier and subsequent infection or replication in underlying target cells. Our studies provide a mechanism by which this common STI could potentially promote the establishment of founder virus populations and the maintenance of local HIV reservoirs in the endocervix. Development of an HIV/STI co-infection model also provides a tool to further explore the role of other sexually transmitted infections in enhancing HIV acquisition.

## Introduction

Women aged 15–24 account for approximately 22% of new human immunodeficiency virus (HIV) infections [1], and heterosexual intercourse is the most common route of transmission in this group [2]. Young women < 25 years of age also have the highest prevalence of *Chlamydia trachomatis* (CT), a sexually transmitted bacterium that can cause adverse reproductive sequelae [3, 4] and is associated with an increased risk of HIV acquisition and increased viral shedding in the female reproductive tract (FRT) of HIV-infected women [5, 6]. CT serovars D-K are obligate intracellular pathogens that infect the columnar epithelial cells of the urogenital tract, and the endocervix is the primary site of infection in women [7]. The endocervix is also a permissive site for sexually transmitted HIV entry [8, 9], suggesting this is a major locale for interactions between the two pathogens. Understanding the mechanisms by which CT could enhance early events in HIV acquisition and replication in target cells at this site would facilitate the design of targeted prevention and intervention strategies to decrease the morbidity associated with both of these pathogens.

CT is the leading bacterial STI in the US and worldwide [4, 10–13], and it has been termed a ‘silent epidemic,’ as it is asymptomatic in >80% of women [14–18]. Asymptomatic women can still display clinical signs of inflammation with one third of patients exhibiting cervicitis upon examination [3, 7]. Chlamydiae can also ascend into the upper reproductive tract where chronic infection can lead to silent or symptomatic inflammation resulting in pelvic inflammatory disease (PID), tubal factor infertility, or ectopic pregnancy. CT undergoes a unique, biphasic developmental cycle that generally modulates between two morphological forms. Extracellular, infectious elementary bodies (EB) attach to epithelial cells, after which they are internalized into a membrane bound vesicle called an inclusion. EBs then differentiate into metabolically active, non-infectious, reticulate bodies (RBs) that undergo binary fission, followed by secondary differentiation into EBs, and are released upon host cell lysis or extrusion [19]. Alternatively, upon nutrient deprivation or stress stimuli, RBs may enter into viable, non-cultivable forms termed persistent bodies (PBs) that can reactivate after the stressor is removed [3, 20–23]. The ability to enter into a persistent state, and other well-documented evasion strategies utilized by the bacteria support natural history studies that indicate CT infections typically last for many months without treatment [3, 20, 24–26]. Further, the natural immunity that is generated in most young women is typically transient, and, therefore, re-infection is common [27, 28]. While routine screening and antibiotic treatment are relatively successful public health strategies that have led to decreased rates of PID in developed countries, many women of low socioeconomic means do not have access to appropriate care, reflecting the necessity for preventative approaches, such as microbicides or a vaccine. Taken together, the chronic, asymptomatic, yet inflammatory, characteristics of clinical chlamydial infections make it likely that CT-infected women would become infected with secondary sexually transmitted pathogens, such as HIV.

The mechanism by which CT infection could enhance HIV acquisition in the FRT has been relatively unexplored. We previously hypothesized that epithelial cells, the primary niche for CT and likely the first cells encountered by luminal HIV in the endocervix, may be central to viral transmission [29]. Potential mechanisms by which CT-infected epithelial cells and their secreted immune mediators could facilitate early HIV transmission events include, but are not limited to: i) disruption of the single columnar endocervical epithelial barrier, facilitating paracellular HIV migration and access to underlying susceptible target cells; ii) enhancement of transcellular HIV migration, also facilitating viral access to underlying susceptible leukocytes; iii) initiation or maintenance of the characteristic influx of activated, CCR5+CD4+ T lymphocytes, the major HIV target cells, observed in the endocervix of CT-infected patients [30, 31], and iv) enhancement of HIV replication in target cells at the site. To address some of these potential mechanisms *in vitro*, we utilized an endocervical epithelial cell line (A2EN) that is easily infected to greater than a 95% infection rate with CT when grown on cell culture inserts [32, 33]. We then used this model to determine whether cell-free or cell-associated HIV could migrate across the CT-infected epithelial barrier and whether CT-infected epithelial cells might play a role in facilitating HIV infection of CCR5+ CD4+ target cells that are present in the submucosa of the endocervix.

## Materials and Methods

### Cells

The A2EN human epithelial cell line was generated in our laboratory from primary endocervical epithelial cells and maintained in Keratinocyte Serum Free Medium (KSFM) supplemented with L-glutamine, calcium, bovine pituitary extract, and epidermal growth factor (Life Technologies, Carlsbad, CA) as previously described [33, 34]. The CCR5+ MT4-R5 T cell line was kindly provided by Dr. James Robinson (Tulane Medical School) and maintained in R10: RPMI1640 containing 10% fetal bovine serum (FBS) and L-glutamine supplemented with 1 μg/ml of puromycin (Invivogen, San Diego, CA) [35]. PM-1 cells [36] and J1.1 Jurkat cells chronically infected with HIV_LAV_ [37] were obtained from the NIH AIDS Reagent Program (ARP) and cultured in R10. TZM-bl cells [38], from the ARP, were maintained in DMEM containing 10% FBS and L-glutamine. Peripheral blood mononuclear cells (PBMC) were isolated from whole heparinized blood (collected from normal donors using an IRB-approved protocol) by density gradient centrifugation on Lymphocyte Separation Medium (MP Biomedicals, Santa Ana, CA). Activated PBMC were generated by stimulating cells with 2.5μg/ml phytohemagglutinin (PHA; Sigma) for 2 d. Both resting and activated PBMC were cultured in RPMI1640 containing 20% FBS, HEPES, penicillin, streptomycin, and L-glutamine (referred to as R20) supplemented with 20 U/ml recombinant human IL-2 (Roche, Basel, Switzerland).

### Virus

The HIV_BaL_ strain [39, 40] was obtained from the ARP. Viral stocks were produced by passaging the virus in either PM-1 cells in R10 or in PHA-activated PBMC in R20 with IL-2. Virus-containing culture medium was harvested at 3 d intervals and clarified by centrifugation (250xg for 10 min) and 0.22μm filtration using a Steriflip apparatus (EMD Millipore, Billerica, MA). The virus was then aliquoted and stored at -80°C. For each stock, the 50% tissue culture infectious dose (TCID_50_) per ml was determined by endpoint titration using TZM-bl cells as described [41]. The HIV_BaL_ passaged in PBMC was used in experiments testing cell-free virus migration as well as testing the effects of CT-infected A2EN cell culture supernatants on HIV infection of PBMC. The PM-1-derived HIV_BaL_ stock was used in all other assays.

### A2EN cell culture on membrane inserts and CT infection

A2EN cells were cultured on 0.4μm pore size polyester Corning Transwell^®^ permeable membrane supports (Corning, NY) and used when their transepithelial electrical resistance (TEERs) exceeded 1000Ω/cm^2^ [33, 42, 43]. Additional wells of A2EN cells grown on cell culture inserts were always seeded in parallel to confirm that monolayers were also impermeable to small molecules using 3kD or 10kD Dextran-conjugated FITC (Sigma, St. Louis, MO) [44–46]. A2EN cells on cell culture inserts were infected with *Chlamydia trachomatis* serovar D (D/UW3/Cx) at an MOI of 3, which yielded a >95% infection rate that could be visualized using fluorescent microscopy, as previously described [32]. Briefly, CT diluted in sucrose-phosphate-glutamic acid buffer (SPG) was placed directly into the cell culture medium in the apical chamber, and cultures were centrifuged for 40 min at 1825xg at 35°C [32]. Infected cells were then incubated for 69 h at 37°C in cell culture medium (without cycloheximide) before the 3 h HIV migration assays were performed, so that experiments were completed at 72 h post chlamydial infection. Mock infections were performed by centrifuging cells in SPG buffer without CT.

### Cell-free and cell-associated HIV migration assays

A2EN cells were infected with CT serovar D. Cell-free HIV_BaL_ inocula of 10, 100, or 1000 TCID_50_ (equivalent to 0.2ng, 2ng, or 20ng p24 or 3 x 10^5^, 3 x 10^6^, or 3 x 10^7^ RNA copies, respectively) was added to the apical chambers of mock or CT-infected A2EN cells for 1, 3, or 24 h at 37°C or 4°C. For 24 h HIV exposure experiments, virus was added to CT-infected A2EN cells at both 48 and 72 h post-CT infection for a total CT infection duration of 72 and 96 h. After the appropriate virus exposure time, the basolateral medium was collected and immediately assessed for infectious HIV using TZM-bl cells or was aliquoted and frozen at -80°C for later analysis of viral content by PCR or p24 ELISA. For cell-associated virus migration assays, HIV-infected cells were first generated by inoculating 2 x 10^7^ MT4-R5 cells with HIV_BaL_ (equivalent to 20ng p24) in puromycin-free R10. After 72 h, the HIV-infected cells were filtered through a 40μm cell strainer, washed twice with Dulbecco’s phosphate buffered saline (DPBS) and resuspended in puromycin-free R10. 1 x 10^6^ HIV-infected MT4-R5 cells were then added to the apical chambers of mock or CT-infected A2EN cells. The basolateral medium was collected 3 h post virus exposure.

### Virus infectivity assays

TZM-bl luciferase reporter cells [38] were used to measure the infectious activity of HIV. For assays evaluating the effects of A2EN supernatants on infectious activity of virus, cell supernatants were incubated for 3 h with 10 TCID_50_ of HIV_BaL_, an amount of virus that produced relative light units (RLU) at least 10-fold above the background in TZM-bl cells. Dilutions of supernatants and virus were then added to TZM-bl cells in triplicate and cultured in black, clear bottom 96 well plates in complete DMEM supplemented with 75μg/mL DEAE-Dextran (Sigma). After 48 h, luminescence was measured as RLU using the Bright-Glo Luciferase Assay System (ProMega, Madison, WI) and a BioTek^®^ Synergy MX plate reader and Gen5^™^ software. For assays evaluating the infectious activity of migrated HIV, 250μl of freshly collected A2EN basolateral supernatant was first incubated with 2 x 10^5^ uninfected MT4-R5 cells to amplify virus. Infectious activity was then assessed 3 and 6 d later by adding 50μl of neat, 1:10, or 1:100 MT4-R5 culture medium to TZM-bl cells in triplicate and measuring RLU 48 h post virus exposure. Negative and positive controls for luciferase expression in TZM-bl cell assays were performed with medium alone or a known amount of HIV_BaL_, respectively.

### Viral RNA quantification

Basolateral supernatants from A2EN cells that had been exposed to cell-free or cell-associated HIV were centrifuged at 20,000×*g* for 1 h at 4°C. The RNA was then purified using Ambion Trizol^®^ Reagent (ThermoFisher, Waltham, MA) in accordance with the manufacturer's instructions. RNA templates were used as standards. The entire sample of RNA from each condition was reverse-transcribed, and polymerase chain reaction (PCR) amplification of the HIV-1 pol gene was performed on each sample, as previously described [47]. Two HIV-negative samples, one HIV-positive sample, and negative experimental controls were included in each assay. HIV levels are reported as copies per replicative sample.

### HIV Infection of PBMC cultured with A2EN supernatants

Resting or PHA-activated PBMC in 100μl of R40 containing 40 U/ml IL-2 were added at 2 x 10^5^ cells per well to a V-bottom 96-well tissue culture plate. An equal volume of epithelial cell medium or undiluted apical or basolateral supernatants from mock or CT-infected A2EN cells was then added. After 2 d at 37°C and 5% CO_2_, the activated PBMC were washed and resuspended in 100μl of medium alone or identical, but freshly thawed, A2EN supernatants. The activated PBMC were then infected with 200 TCID_50_ HIV_BaL_ in 100μl of R40 with 40 U/ml IL-2. The resting PBMC were washed and infected with 200 TCID_50_ HIV_BaL_ in 0.2ml of R20 with 20 U/ml IL-2 after 4 d. On d2 and d4 after infection, the culture medium was completely replaced with 200μl of R20 containing 20 U/ml IL-2. Culture medium harvested on day 6 was treated with Triton X-100 (0.5% v/v) and analyzed for viral content using a p24 ELISA.

### p24 ELISA

Concentrations of p24 were measured using a p24 ELISA, as previously described [48]. Briefly, high protein binding microtiter plates (ThermoFisher) were coated overnight with an anti-p24 monoclonal antibody that had been purified from H12 hybridoma (ARP) culture medium using Protein G Sepharose (ThermoFisher). Plates were washed with PBS containing 0.05% Tween-20 (PBST) and blocked with PBST containing 5% nonfat dry milk and 1% FBS. Samples were serially diluted in 1% FBS/PBST and added to plates. A recombinant HIV p24 protein (ImmunoDiagnostics, Woburn, MA) previously calibrated using the PerkinElmer HIV p24 ELISA kit was used to generate a standard curve. Following an overnight reaction at 4°C, the plates were washed and treated for 1 h at 37°C with HIVIG (ARP) that had been biotinylated in the lab using EZ Link Sulfo NHS (ThermoFisher). The plates were then washed and consecutively treated for 30 min at room temperature with neutralite avidin-peroxidase and tetramethylbenzene (both SouthernBiotech, Birmingham, AL). After addition of H_2_SO_4_ stop solution, absorbance was recorded at 450nm. The concentration of p24 in samples was then interpolated from standard curves constructed with the SoftMax Pro computer program (Molecular Devices, Sunnyvale, CA).

### Statistical Analyses

Statistical analyses were performed using Prism software (v4.0; GraphPad, San Diego, CA). One-way analysis of variance (ANOVA) with Tukey post-test was used to compare results in most experiments. One-tailed Mann-Whitney rank sum test was used to analyze cumulative p24 results from HIV-infected PBMCs cultured with apical or basolateral epithelial supernatants collected in 3 separate experiments. P-values <0.05 were considered significant.

## Results

### A2EN cells grown on cell culture inserts create a tight barrier against cell-free HIV

**Fig 1:**
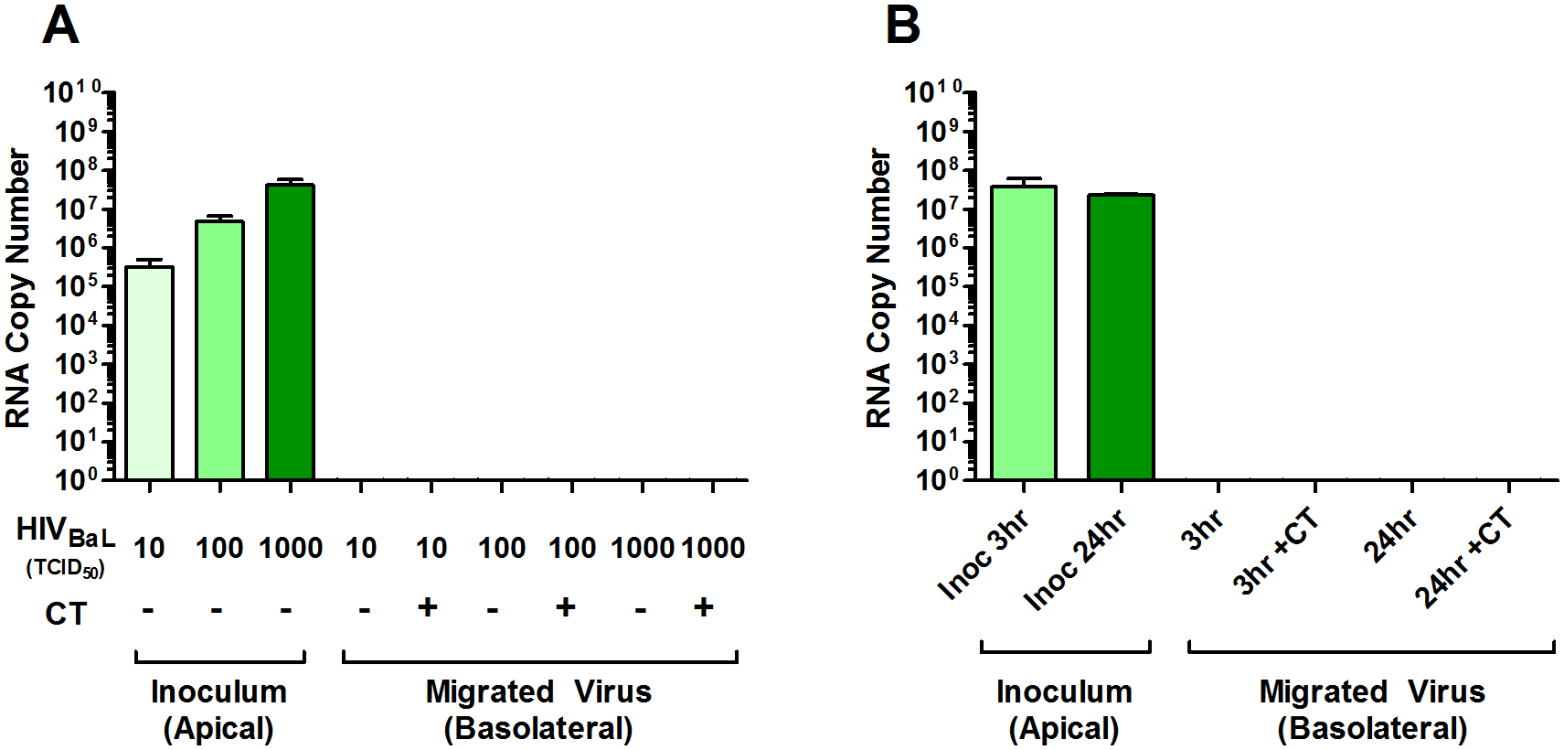
Cell-free HIV does not migrate across mock or CT-infected A2EN cells. (**A**) A2EN cells were infected with CT serovar D (> 95% infection rate) for 71 h as described in Materials and Methods. 10, 100, or 1000 TCID_50_ of cell-free HIV_BaL_ was added to the apical chambers of mock and CT-infected A2EN cells for 1 h at either 4°C or 37°C so that the CT infection duration was 72 h total. (**B**) 1000 TCID_50_ of cell-free HIV_BaL_ was added to the apical chambers of mock and CT-infected A2EN cells for 3 or 24 h at 37°C for a total CT infection duration of 72 h. Basolateral supernatants were then collected and analyzed for viral RNA using quantitative real-time PCR. Bars represent the mean values of RNA copies ± the standard deviation (SD). Data is representative of 4 independent experiments, each performed in quadruplicate. *p < 0.05.

Both cell-free and cell-associated HIV have been detected in genital secretions [49, 50], and both are considered important modalities for sexual transmission of HIV [51–59]. We first determined whether cell-free virus could traverse from the apical surface of an A2EN monolayer into the basolateral compartment. The CCR5-tropic HIV_BaL_ strain was selected for these studies, as sexually transmitted HIV isolates are predominantly CCR5-tropic [60–64]. Cell-free virus migration was investigated using varying inocula of virus, ranging from 10 TCID_50_, an amount at the high range of a physiological inoculum [65, 66], up to 1000 TCID_50_, an amount that others have reported is necessary for observing HIV transcytosis [54–56]. Cell-free virus was added to the apical chamber of A2EN cells for 1 h at either 37°C or 4°C in order to differentiate between transcellular and paracellular virus migration. Basolateral supernatants were collected and analyzed using a highly sensitive, quantitative PCR assay that can accurately detect as few as 10 copies of intact viral RNA per PCR reaction, as previously described [47]. We chose to use this assay to confidently measure HIV virions that cross the epithelial barrier rather than measuring HIV capsid protein, as p24 may be present on defective viral particles, cross the epithelium, and confound migration results. No viral RNA was detected in the basolateral chambers of either mock or CT-infected A2EN cells after 1 h at either 37°C (Fig 1A) or 4°C (data not shown) using any concentration of virus. We then used the highest inoculum (1000 TCID_50_), and increased the incubation times to 3 and 24 h, the latter of which is significantly longer than exposure times used in any other reports of cell-free transcytosis through an epithelial cell monolayer [54, 67–71]. For 24 h HIV exposure experiments, virus was added at both 48 and 72 h post-CT infection for a total CT infection time of 72 and 96 h (96h total duration time not shown), and no significant host cell lysis was observed, as previously described [32], making paracellular migration of virus in this assay an unlikely event. Even after 24 h, no viral RNA was observed in the basolateral chamber of either mock or CT-infected A2EN cells (Fig 1B), indicating that essentially no cell-free virus migrated through the epithelium.

It is possible, however, that virus migrated across the A2EN barrier at such a low rate that it was below the detection limit of our PCR assay. To investigate this, basolateral supernatants from A2EN cells exposed to 1000 TCID_50_ cell-free HIV_BaL_ were incubated with uninfected CCR5+ MT4-R5 cells to amplify any viable virus that might have been in the basolateral supernatants. After 6 d, the MT4-R5 culture medium was tested for the presence of infectious HIV using TZM-bl cells. No virus was detected (data not shown). Overall, these results suggest that A2EN cells provide a tight, impenetrable barrier against cell-free HIV, similar to findings of others using different epithelial cells [55, 56, 67, 72, 73]. Furthermore, CT infection of A2EN cells did not modulate the integrity of the epithelial barrier to allow passage of cell-free HIV via either transcellular or paracellular mechanisms.

### CT infection of A2EN cells enhances cell-associated HIV migration

**Fig 2:**
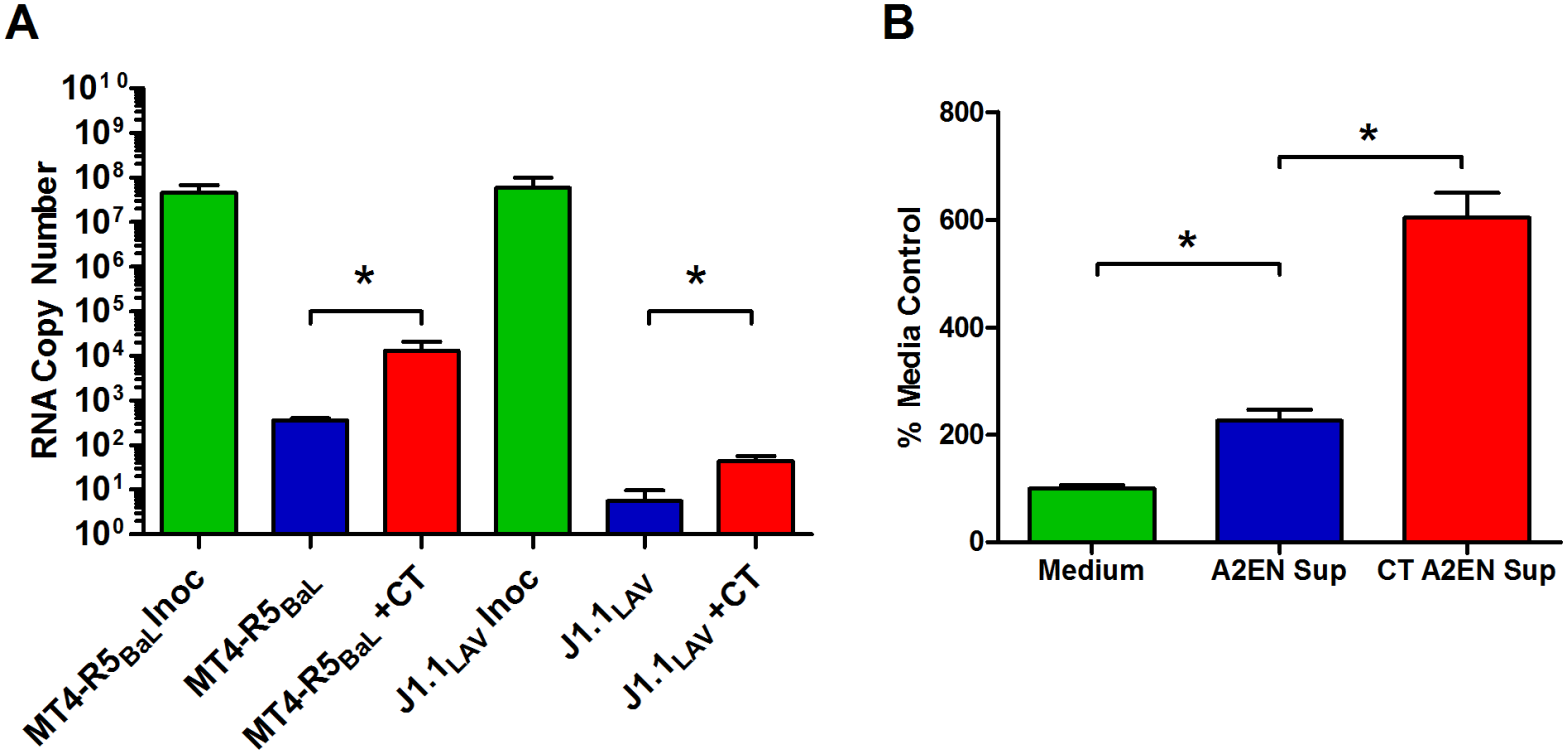
Cell-associated HIV crosses the A2EN epithelial barrier and is enhanced by CT infection. A2EN cells were infected with CT serovar D (> 95% infection rate) for 72 h. 1 x 10^6^ HIV_BaL_-infected MT4-R5 cells were added to the apical chambers of mock and CT-infected A2EN cells for 3 h at 37°C. (**A**) Viral RNA in basolateral supernatants from cell-associated HIV migration assays was quantitated using real-time PCR. Bars represent the mean RNA copies ± SD. (**B**) Basolateral supernatants collected in cell-associated HIV migration assays were incubated with uninfected MT4-R5 cells for 3 d. Supernatants from exposed MT4-R5 cells were then assayed for infectious virus using TZM-bl cells. Controls included TZM-bl cells incubated with an equivalent volume of epithelial cell medium. Data is expressed as a percentage of the mean RLU obtained for the medium alone controls. Bars represent the mean percentages ± SD. Results shown are representative of 4 separate experiments, each performed in quadruplicate. *p < 0.05.

Next, we determined if cell-associated HIV could migrate across the A2EN monolayer. We first developed a standardized, reproducible protocol that generated a productive infection of MT4-R5 cells with HIV_BaL_, and consistently yielded RNA copies/ml similar to those in the 1000 TCID_50_ inoculum used above in experiments with cell-free HIV. 1 x 10^6^ HIV_BaL_-infected MT4-R5 cells were added to mock and CT-infected A2EN cells for 3 h. Basolateral supernatants were collected and the viral RNA was quantified. Importantly, cell-associated HIV crossed the A2EN barrier at low levels (approximately 362 mean viral RNA copies per well), and these levels were significantly increased (approximately 13,264 mean viral RNA copies per well) when A2EN cells were infected with CT (p < 0.05; Fig 2A). These results were confirmed using a p24 ELISA (data not shown). To determine whether the migrated virus was infectious, basolateral supernatants were collected 3 h after virus exposure and incubated with uninfected MT4-R5 cells. Cultures were tested for infectious virus at 3 and 6 d (3 d time point shown in Fig 2B) using TZM-bl cells. Viable virus was detected at both time points, confirming that migrated virus was infectious. Migration assays were also performed with J1.1 Jurkat T cells chronically infected with the HIV_LAV_ CXCR4-tropic virus. Similar findings were observed using J1.1 cells in mock and CT-infected A2EN cells (Fig 2A), suggesting that neither cell-associated HIV migration nor chlamydial enhancement of this migration was dependent upon viral tropism (CXCR4 vs CCR5). Taken together, these data suggest that HIV-infected T cells may facilitate low levels of virus migration across the endocervical epithelium, and that CT infection may enhance this process.

### HIV-infected MT4-R5 cells do not significantly compromise the integrity of the A2EN epithelial barrier

**Fig 3:**
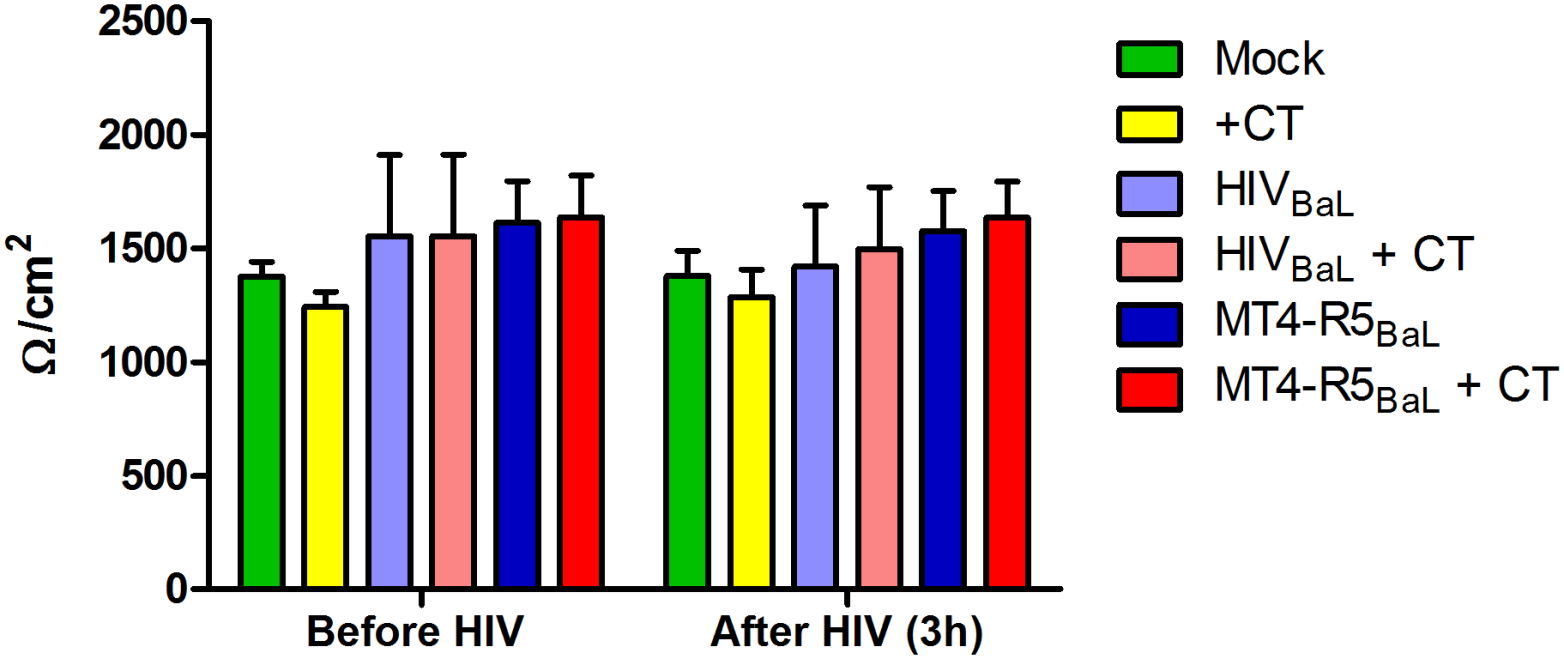
A2EN epithelial cell barrier integrity is not compromised by CT, cell-free HIV, or HIV-infected cells. A2EN cells on cell culture inserts were infected with CT serovar D for 72 h. 1000 TCID_50_ of cell-free HIV_BaL_ or 1 x 10^6^ HIV_BaL_-infected MT4-R5 cells were then added to the apical surface of mock or CT-infected cells for 3 h. TEERs were measured before and after the addition of virus or infected cells. Bars represent the mean TEER values ± SD pooled from 3 separate experiments, each performed in triplicate.

One mechanism by which cell-associated HIV could cross an epithelial monolayer is by paracellular migration as a result of epithelial barrier disruption. Although we previously reported that chlamydial infection of A2EN cells grown on cell culture inserts did not decrease the epithelial barrier integrity [32], it is possible that the addition of HIV-infected T cells could disrupt the epithelium and allow virus to diffuse through the monolayer. To ascertain this, TEERs of both mock and CT-infected A2EN cells were recorded before and after the 3 h incubation with cell-free virus or infected MT4-R5 cells (Fig 3). There were no significant differences between TEERs, suggesting that neither cell-free nor cell-associated virus grossly disrupted junctional complexes or the tight barrier formed by A2EN epithelial cells. FITC-labeled dextran (3kD and 10kD) was also utilized to confirm that small molecules were incapable of penetrating A2EN cells after CT, HIV, or dual pathogen exposure (data not shown). These results suggest that the virus migration observed using HIV-infected MT4-R5 or J1.1 T cells was not due to disruption of the CT-infected A2EN epithelial barrier.

### HIV migration is likely dependent upon epithelial cell contact with HIV-infected T cells

**Fig 4:**
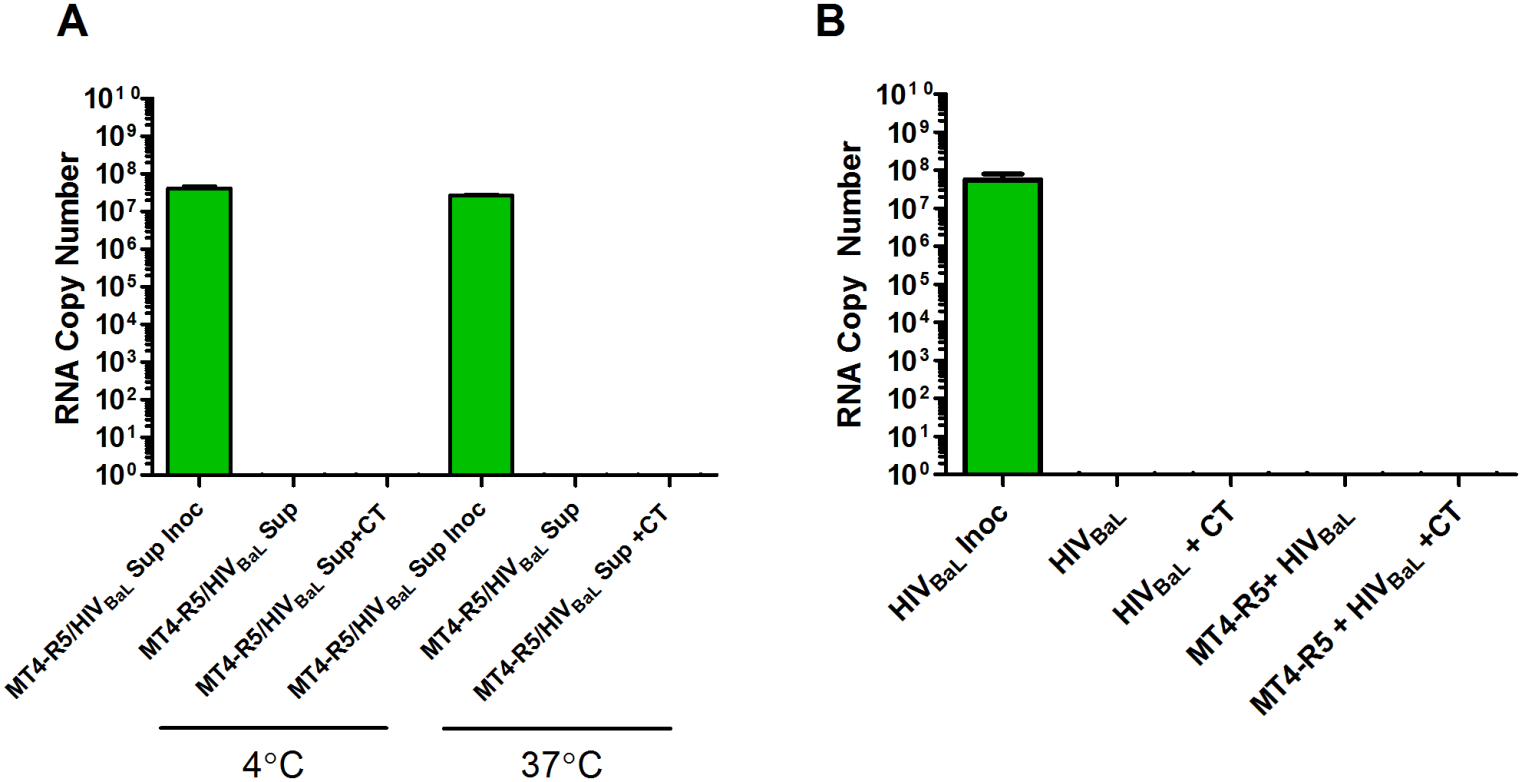
Cell-associated HIV_BaL_ migration is likely dependent upon epithelial cell contact with an HIV-infected T cell. A2EN cells were infected with CT serovar D for 72 h. (**A**) 1000 TCID_50_ of cell-free HIV_BaL_ freshly produced by MT4-R5 was added to apical chambers of mock and CT infected cells for 3 h at 4°C or 37°C. (**B**) 1000 TCID_50_ of PM-1-derived cell-free HIV_BaL_ was added to mock and CT-infected A2EN cells in the presence or absence of 1 x 10^6^ uninfected MT4-R5 cells. After a 3 h at 37°C, basolateral supernatants were collected and viral RNA was enumerated using PCR. Bars represent the mean RNA copies ± SD. Data is representative of 3 independent experiments, each performed in quadruplicate. *p < 0.05.

It is possible that cell-associated HIV crossed the A2EN barrier more efficiently than cell-free virus simply because the MT4-R5 cell line produced “fitter, more infectious virus” than PM-1 cells, the cell line in which the cell-free HIV_BaL_ had been generated. Host adhesion molecules incorporated into the viral envelope might also differ for the HIV_BaL_ produced in these cell lines [74, 75]. To confirm that MT4-R5-derived virus did not behave differently than PM-1-derived virus, 1000 TCID_50_ of cell-free HIV_BaL_ freshly produced by HIV_BaL_-infected MT4-R5 cells was added to the apical chambers of mock and CT-infected A2EN cells. No viral RNA was observed in the basolateral chambers (Fig 4A), suggesting that virus produced by MT4-R5 cells does not cross the tight A2EN barrier more efficiently. A 1000 TCID_50_ dose of HIV_BaL_ produced in PBMC also failed to cross the CT-infected A2EN epithelial barrier (data not shown).

It is also possible that cell-associated virus migrated more efficiently because the T cells contacted the epithelial cells and stimulated specific cellular signaling events, making the epithelial cells more susceptible to virus entry [55, 58, 67, 76]. To determine if this might be the case, cell-free HIV_BaL_ and uninfected MT4-R5 cells were simultaneously added to the apical chamber of mock and CT-infected A2EN cells. Consistently, we failed to observe any viral RNA in the basolateral chamber when uninfected T cells contacted the epithelial cells in the presence of cell-free virus (Fig 4B). These and the above results suggest that epithelial contact with an HIV-infected T cell is likely required for HIV migration across the A2EN epithelial cell barrier.

### A2EN cell supernatants do not decrease infectious activity of HIV

**Fig 5:**
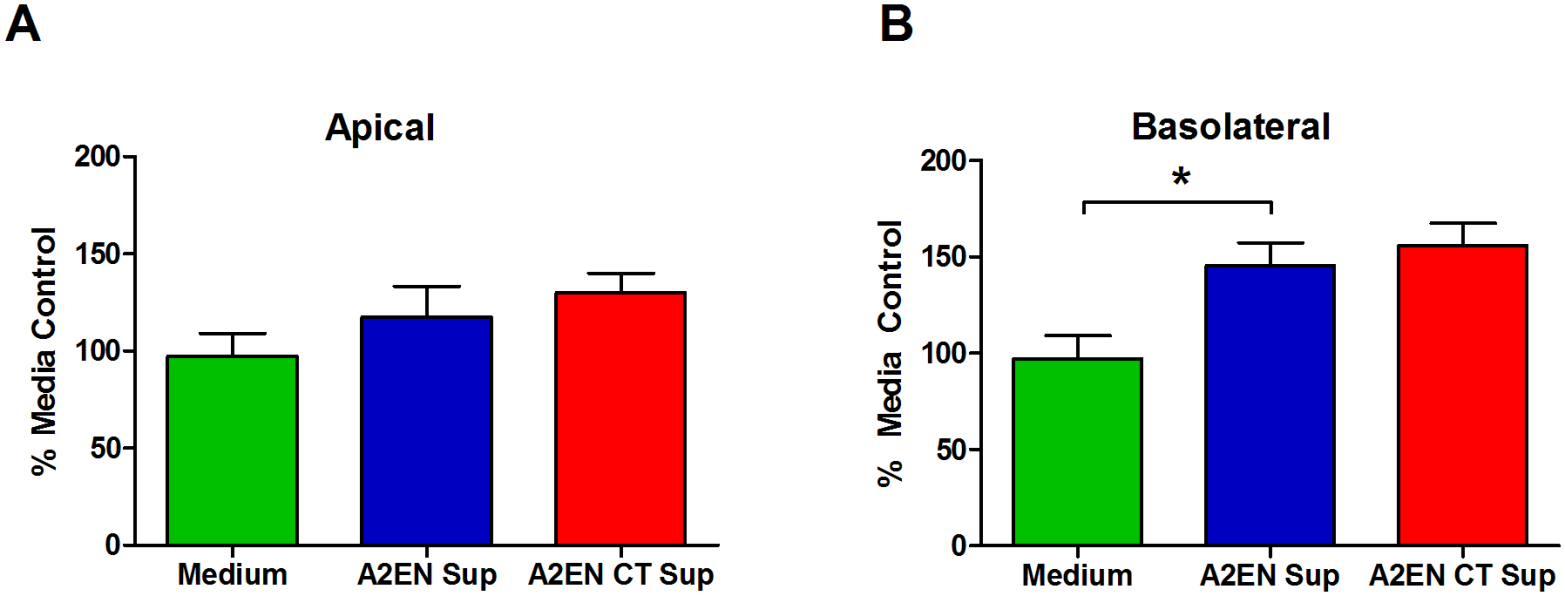
A2EN supernatants do not decrease cell-free HIV viability. A2EN cells were infected with CT serovar D, and supernatants were collected 72 h post-infection. Medium alone, (**A**) apical, or (**B**) basolateral supernatants were incubated with 1000 TCID_50_ of cell-free HIV_BaL_ for 3 h. The infectious activity of the virus was then measured by transferring to TZM-bl cells and measuring luciferase activity 48 h later. Data shown is expressed as a percentage of the mean RLU recorded for medium alone controls. Bars represent the mean percentages ± SD. Data is representative of 3 independent experiments, each performed in quadruplicate. *p < 0.05.

It is possible that cell-free HIV migration was not observed because anti-microbial molecules in A2EN supernatants neutralized cell-free HIV at the apical surface and prevented viral migration [77–81]. CT infection has been shown to increase anti-microbial peptide expression in the reproductive tract and endocervical epithelial cells ([82]; unpublished observations), which might further decrease the probability that viable cell-free virus could cross the epithelium. To investigate this, apical or basolateral supernatants collected from mock or CT-infected A2EN cells were cultured with HIV_BaL_ for 3 h. The infectious activity of the virus was then assessed using TZM-bl cells. There were no significant decreases in viral infectivity after culture with apical supernatants (Fig 5). Interestingly, basolateral supernatants from both mock and CT-infected A2EN cells increased viral infectivity above the medium control (p<0.05; Fig 5B). These results suggest that neither apical or basolateral A2EN supernatants, nor CT-infected A2EN supernatants, decrease HIV viability. Thus, a reduction in virus viability in the apical compartment does not explain why cell-free virus did not cross either the mock or CT-infected A2EN epithelial barrier. Furthermore, A2EN may produce molecules that enhance HIV infectivity of susceptible cell types.

### CT infection of A2EN cells results in an enhancement of HIV infection of target cells

**Fig 6:**
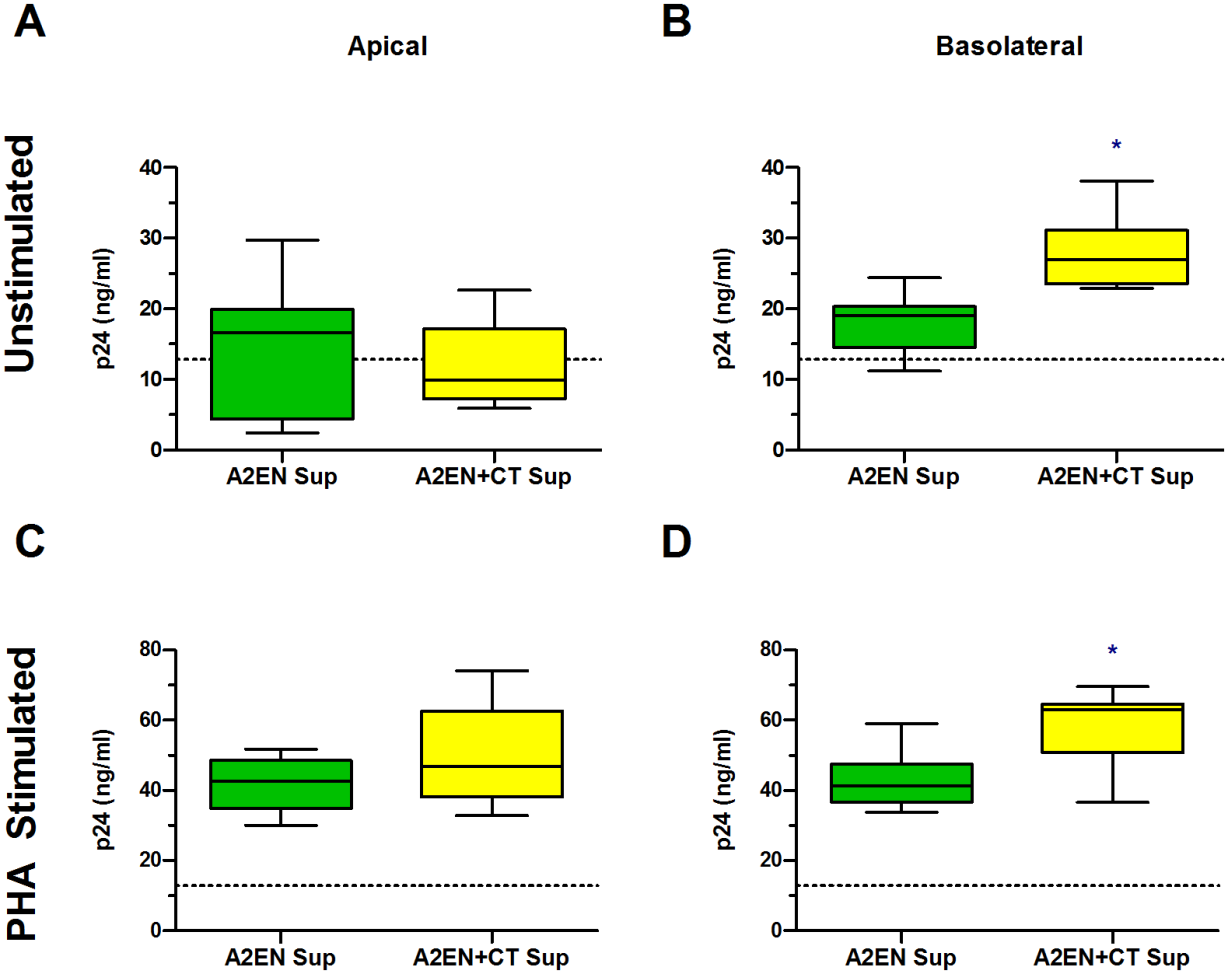
Basolateral supernatants from CT-infected A2EN cells enhance HIV infection of PBMC. A2EN cells grown on cell culture inserts were infected with CT serovar D for 72 h. (**A, C**) Apical and (**B, D**) basolateral supernatants collected from mock or CT-infected A2EN cells were incubated with (**A, B**) resting PBMC for 4 d or (**C, D**) PHA-activated PBMC for 2 d. The PBMC were then infected with 200 TCID_50_ of HIV_BAL_. Activated PBMC were additionally cultured with A2EN supernatants for another 2 d after infection. Every 2 d after infection, the culture medium was completely replaced. The viral content in culture medium collected 6 d after infection was determined using a p24 ELISA. Presented are the cumulative p24 results from 3 separate experiments, each performed in triplicate. Bars represent the mean ± SD. *p < 0.05.

We previously established that CT infection of A2EN cells significantly upregulates IL1α, a pro-inflammatory cytokine that can activate NFκB and could increase virus replication in target cells [32]. Concomitantly, CT infection abrogates the secretion of the CCR5 agonist RANTES, which would increase the chance that HIV could bind to its co-receptor on target cells [32]. Based on this and the above results showing enhanced HIV infection of TZM-bl cells in the presence of basolateral cell supernatants (Fig 5), we hypothesized that secretions from CT-infected endocervical epithelial cells may enhance HIV entry and/or replication in CCR5+ HIV target cells. To investigate this, PHA-activated PBMC were incubated with epithelial cell medium alone or apical or basolateral supernatants from mock or CT-infected A2EN cells for 2 days prior and 2 days after infection with HIV_BaL_. Viral production by PBMC was measured 6 d after HIV infection using a p24 ELISA that can accurately distinguish smaller fold changes in HIV compared to our qPCR assay (Fig 6). We also chose to test resting PBMC because we wanted to investigate the possibility that supernatants from CT-infected A2EN cells could partially activate resting T cells, making them more susceptible to HIV infection. For these assays, resting PBMC were pre-cultured with KSFM medium or A2EN supernatants for 4 d prior to infection with HIV_BaL_. Basolateral secretions from mock-infected A2EN cells were found to increase viral production over medium alone controls by approximately 2-fold in both resting and activated PBMC (p < 0.05, p24 levels of medium alone controls of resting and PHA stimulated PBMCs were 7.02 and 26.85pg/mL, respectively). However, exposure of resting or activated PBMC to basolateral supernatants from CT-infected A2EN cells increased viral production to a greater extent (Fig 6B).

Similar results were obtained when TZM-bl or MT4-R5 cells were cultured with A2EN supernatants for 4 d, then infected with PM-1-derived HIV_BaL_ (S1 Fig). Taken together, these observations suggest endocervical epithelial cells may create a milieu at the basolateral surface that may activate resting T cells and facilitate HIV entry and/or replication in activated target cells. Should HIV gain access to the submucosa via trauma or a migration mechanism, CT-infected endocervical epithelial cells may produce factors that enhance HIV infection of target cells, potentially aiding in the establishment of early founder virus populations.

## Discussion

HIV transmission studies using high dose, vaginal, cell-free SIV in non-human primate models, report that virus can cross the endocervical epithelium leading to recruitment of target cells and formation of small foci of infected T lymphocytes in the cervical stroma [8, 9, 83]. Clinical, diagnostic, and basic scientific studies, together, also demonstrate that endocervical epithelial cells are the major niche for CT, they produce proinflammatory cytokines in response to the organism, and infection is commonly accompanied by an influx of leukocytes, including activated, memory and CCR5+ T cells [30, 31]. Considering that these leukocyte subsets are the major targets for sexually transmitted strains of HIV [7, 30, 31], collectively, these observations suggest that an existing endocervical chlamydial infection could increase the likelihood of HIV transmission at this site and indicate a central role for infected epithelial cells as facilitators in this process. We therefore developed an endocervical epithelial cell model, A2EN, to investigate the direct contribution of CT infected epithelial cells in enhancing HIV migration and replication in target cell populations found in the submucosa of the CT infected endocervix.

We were able to demonstrate that CT infection of A2EN cells enhanced cell-associated, but not cell-free, virus migration across the epithelial barrier. These data support earlier studies which reported that cell-associated HIV is present in the in semen of infected men and may gain access to subepithelial target cells in the FRT of women [49, 51–53, 84], and it suggests that CT infection may further enhance this process. We also observed enhanced HIV infection in PBMC and CD4+, CCR5+ cell lines exposed to basolateral supernatants from CT-infected epithelial cells. These latter results suggest that, should virus cross the endocervical epithelial barrier, CT infection may facilitate viral infection in the local microenvironment, enhancing the probability of establishing a productive HIV infection.

Previous studies, using a variety of modeling conditions, have been conflicted as to whether cell-free HIV could traverse across an intact epithelium [49, 51, 52, 54–59, 69, 70, 72, 84–91]. Here, we focused on how HIV migrates across a mucus-producing, single cell, endocervical epithelial cell layer that recapitulates many primary properties of the endocervix [33, 34]. We were unable to observe any cell-free HIV migration across A2EN cells, suggesting that the epithelial barrier was not significantly compromised upon exposure to either CT or HIV. Furthermore, we were unable to observe any differences in epithelial barrier function between TEERs or dextran permeability in CT-infected, HIV-exposed A2EN cells, making disruption of tight junctions and subsequent paracellular migration of cell-free HIV an unlikely mechanism in this model. We also verified that the lack of cell-free HIV migration was not due to neutralization of virus by apically secreted anti-microbial molecules, a finding in contrast to others [77–81]. We utilized a high concentration of virus to ascertain viral migration and infectivity, which may account for the differences in our study compared to others [54, 67, 69–71]. It cannot be ruled out, however, that antimicrobial molecules may have been concentrated in the mucus on the apical surface of A2EN cells [92–94] and not sufficiently solubilized in the cell culture supernatants, which may have influenced HIV binding, viability, and/or entry. Another possible reason why paracellular cell-free HIV migration did not occur may be because A2EN cells infected with CT or exposed to HIV did not secrete TNFα at sufficient levels to disrupt tight junctions [32], which has been shown to occur in endometrial epithelial cell models [95]. The differences between our results and those of Nazli *et al*. [95] may highlight tissue-specific differences in the innate immune responses to HIV and subsequent virus diffusion across the epithelial barrier. Experiments using endocervical and endometrial epithelial cells derived from the same donor should ideally be performed to specifically evaluate these different sites and their roles in the immune response, their barrier functions and permissiveness for HIV migration, as all of these could influence the efficacy of preventative microbicides or vaccines against CT and HIV in the FRT. Moreover, the role of leukocyte-epithelial interactions should also be investigated within these model systems, as the dynamics of these interactions may also significantly and differentially influence parameters affecting early HIV transmission events.

While we were unable to detect cell-free HIV migration, cell-associated virus crossed the endocervical epithelial barrier with low efficiency, and this significantly increased in the presence of chlamydial infection. These results support other laboratories’ work demonstrating an important role for cell-associated virus in HIV acquisition in the FRT [51–53, 55, 84, 96]. The low efficiency rate observed by us and others may aid in explaining why heterosexual transmission rates are low, as the epithelium, mucus, and secreted antimicrobial molecules in the FRT clearly can create an effective barrier to prevent high numbers of virions from gaining access to the submucosa. Despite the fact that many have observed that cell-associated virus can migrate across epithelial barriers [55, 58, 67, 76, 85, 97], the mechanism of how this occurs is still unknown. The cell-associated viral migration observed in our study was not simply due to ‘fitter’ virus or T cell contact, suggesting that there may be a “synapse” that occurs between HIV infected lymphocytes and epithelial cells that facilitates the uptake and/or migration process, a mechanism that has been proposed by others [67, 87]. CT infection of A2EN cells may enhance this “synaptic” process, allowing more virions to gain access to the basolateral compartment. Since neither TEERs nor dextran permeability significantly changed upon the addition of HIV-infected T cells to A2EN cells, it is likely that cell-associated virus migration occurred via a trans-cellular mechanism, such as the endocytic recycling pathway [88], as others have reported. It is also possible that cell-associated virus may cross via a paracellular mechanism if HIV-infected T lymphocytes extend pseudopodia between the epithelial cells without significantly disrupting the tight junctions, which can occur with HIV-infected dendritic cells and intestinal epithelial cells [98]. Virus may also cross the epithelial cell barrier by a sequestration process whereby virions are taken up at the apical surface and released at the basolateral membrane non-specifically [89, 90, 96, 99, 100]. Distinguishing sequestration from an active transcytosis mechanism is challenging as it is not yet known what host cellular machinery is utilized by HIV to facilitate either process. Overall, HIV and CT may modulate multiple factors in the endocervical epithelial cells that could facilitate cell-associated virus migration. Further, in depth, experimentation might be warranted to elucidate the exact mechanisms involved in order to design specific therapies to inhibit these initial HIV infection events.

We previously reported that CT infection of A2EN cells significantly upregulated the proinflammatory cytokine, IL1α, but abrogated the expression of the CCR5 ligand RANTES [32]. Taken together, we hypothesized that supernatants from CT-infected A2EN may enhance HIV replication in target cells, as IL1α can stimulate the NFκB pathway in target cells, leading to increased HIV replication [101, 102]. Concomitantly, a decrease in RANTES could allow more virions to bind to the CCR5 co-receptors present on target cells [103–105]. When basolateral supernatants from non-infected A2EN cells were incubated with PBMC or MT4-R5 cells and inoculated with virus, we observed an increase in HIV production. Furthermore, CT-infected A2EN basolateral supernatants further enhanced HIV production, but apical supernatants did not, despite the finding that apical IL1α increases after chlamydial infection [32]. These results may be explained by the fact that A2EN cells secrete IL1 receptor antagonist (IL1ra) at the apical surface, but not at the basolateral surface [32], which would inhibit IL1α from functioning in the apical environment. Our results suggest that the functional IL1α, or an unidentified factor, or a combination of factors present in the basolateral supernatants from CT-infected A2EN cells, may be acting upon target cells to facilitate HIV entry and/or replication. Should HIV gain access to the endocervical submucosa via migration or trauma, virus may be more likely to infect and replicate in an underlying susceptible target cell. Considering these results and our previous observations that CT infection of the endocervix leads to an increase in CD4+ and CCR5+ lymphocytes at this site [30, 31], the microenvironment created by CT-infected epithelial cells may contribute to the establishment of HIV founder virus populations in target cells present within the cervical submucosa.

In conclusion, our endocervical model is one of the first to allow the *in vitro* study of interactions between the two sexually transmitted pathogens, CT and HIV. It could also serve as a tool for similar co-infection models, microbicide testing, screening of specific neutralizing antibodies, or for building more complex models that incorporate various defined leukocyte subpopulations. Further, the tractability and primary cell-like properties of the model could enable the investigation of endogenous and exogenous cofactors such as semen, mucus, and hormones and their influence on cell-free and cell-associated HIV transmission. The studies presented here suggest CT infection of endocervical epithelial cells increases cell-associated HIV migration across the epithelial barrier and can enhance viral replication in target cells via basolaterally-secreted factors. These results suggest CT infection of endocervical epithelial cells could facilitate HIV access to underlying susceptible cell types, the establishment of a founder virus population, and ultimately, HIV transmission. The ability of CT to infect the FRT for extended periods of time in the absence of symptoms likely increases the probability of introducing a secondary pathogen, such as HIV, into a CT-infected FRT. Collectively, these observations reiterate the importance of finding alternative preventative strategies for CT infection, as this and other highly prevalent sexually transmitted pathogens contribute to HIV acquisition in women worldwide.

## Supporting Information

S1 Fig Basolateral fluids from mock and CT-infected A2EN cells enhance HIV infection. A2EN cells on cell culture inserts were infected with CT serovar D. Supernatants from mock or CT-infected A2EN cells were collected after 72 h and incubated with either TZM-bl cells or MT4-R5 cells for 24 h. 10 TCID_50_ was then added to each well of TZM-bl cells or to 2 x 10^5^ MT4-R5 cells. (**A, B**) Virus exposed TZM-bl cells were assayed for luciferase expression 48 h after virus addition. (**C, D**) Virus exposed MT4-R5 T cells were incubated for 6 d. The culture medium was then tested for infectious HIV after transfer to TZM-bl cells. Controls included TZM-bl cells incubated with a known amount of virus or an equivalent volume of KFSM medium alone. Data shown is expressed as percentage of the mean RLU obtained with KFSM controls. Bars represent the mean percentages ± SD. Data is representative of 3 independent experiments, each performed in quadruplicate. *p < 0.05.
(TIF)
