# Supplementary figures and images for: Chlamydia trachomatis Infection of Endocervical Epithelial Cells Enhances Early HIV Transmission Events
Source: PLoS One. 2016 Jan 5;11(1):e0146663. doi: 10.1371/journal.pone.0146663 (PMC4701475; doi:10.1371/journal.pone.0146663)

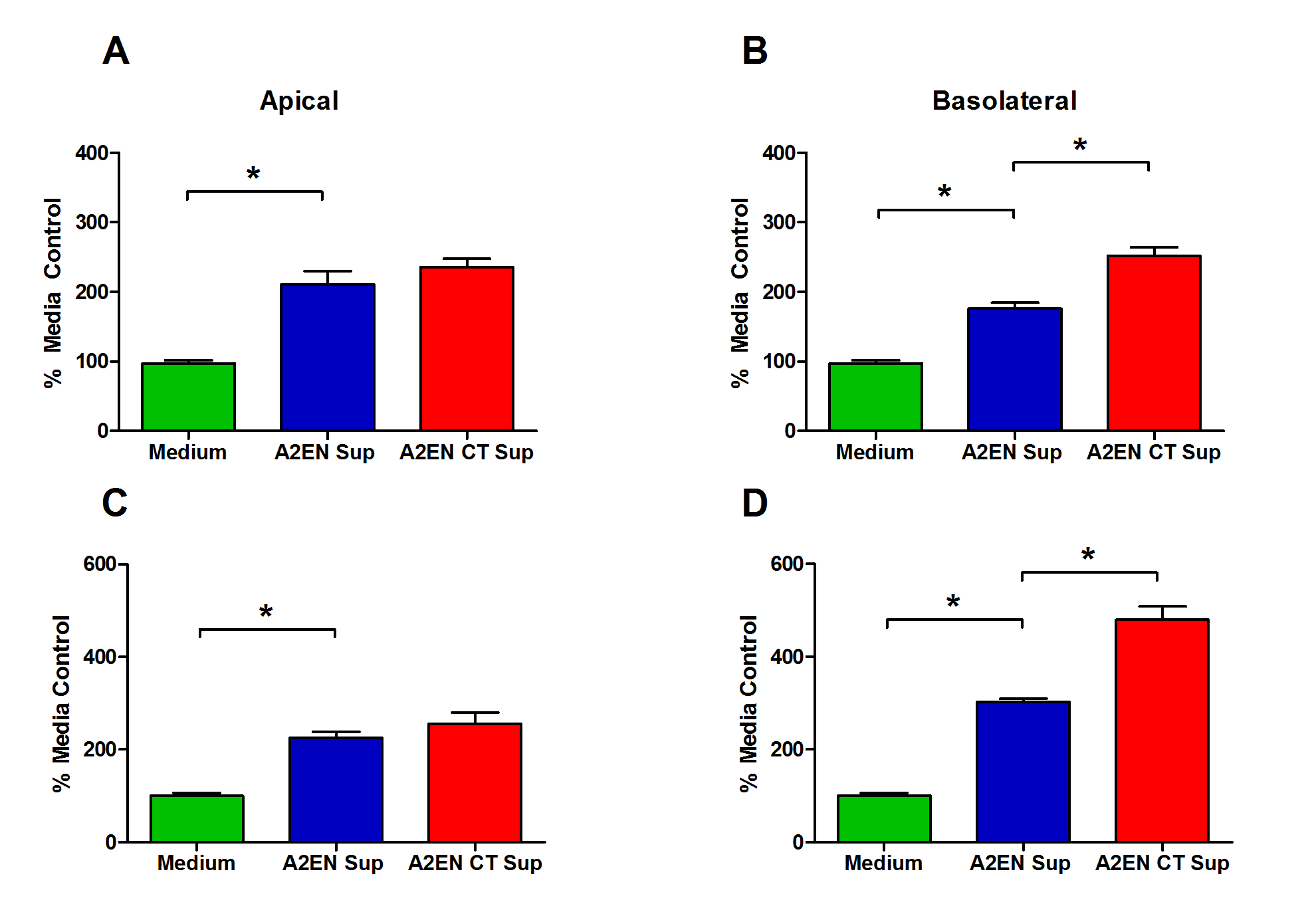

Supplement: S1 Fig — A2EN cells on cell culture inserts were infected with CT serovar D. Supernatants from mock or CT-infected A2EN cells were collected after 72 h and incubated with either TZM-bl cells or MT4-R5 cells for 24 h. 10 TCID50 was then added to each well of TZM-bl cells or to 2 x 10^5^ MT4-R5 cells. (A, B) Virus exposed TZM-bl cells were assayed for luciferase expression 48 h after virus addition. (C, D) Virus exposed MT4-R5 T cells were incubated for 6 d. The culture medium was then tested for infectious HIV after transfer to TZM-bl cells. Controls included TZM-bl cells incubated with a known amount of virus or an equivalent volume of KFSM medium alone. Data shown is expressed as percentage of the mean RLU obtained with KFSM controls. Bars represent the mean percentages ± SD. Data is representative of 3 independent experiments, each performed in quadruplicate. *p < 0.05. (TIF) [file pone.0146663.s001.tif]
